# Expression of Concern to: Diet restriction inhibits apoptosis and HMGB1 oxidation and promotes inflammatory cell recruitment during acetaminophen hepatoxicity

**DOI:** 10.1186/s10020-020-0140-z

**Published:** 2020-01-30

**Authors:** Daniel James Antoine, Dominic P. Williams, Anja Kipar, Hugh Laverty, B. Kevin Park

**Affiliations:** 1 Medical Research Council Centre for Drug Safety Science, Liverpool, UK; 2 Department of Pharmacology and Therapeutics, Institute for Translational Medicine, Liverpool, UK; 3 Veterinary Pathology, School of Veterinary Science, University of Liverpool, Liverpool, UK

## Abstract

The Editors-in-Chief would like to alert readers that this article [1] is part of an investigation being conducted by the journal following the conclusions of an institutional enquiry at the University of Liverpool with respect to the quantitative mass spectrometry-generated results regarding acetylated and redox-modified HMGB1.

**Expression of Concern to: Mol Med (2010) 16:479-490**

**https://doi.org/10.2119/molmed.2010.00126**

The Editors-in-Chief would like to alert readers that this article (Antoine et al., 2010) is part of an investigation being conducted by the journal following the conclusions of an institutional enquiry at the University of Liverpool with respect to the quantitative mass spectrometry-generated results regarding acetylated and redox-modified HMGB1. Appropriate editorial action will be taken once the investigation is concluded.

Dominic P. Williams, Anja Kipar, and Hugh Laverty agree to this editorial expression of concern.

B. Kevin Park and Daniel J. Antoine have not responded to any correspondence from the editor/publisher about this editorial expression of concern.
